# Bottlenecks for genome-edited crops on the road from lab to farm

**DOI:** 10.1186/s13059-018-1555-5

**Published:** 2018-10-26

**Authors:** Armin Scheben, David Edwards

**Affiliations:** School of Biological Sciences and Institute of Agriculture, The University of Western Australia, Perth, WA 6009 Australia

## Abstract

Gene discovery and government regulation are bottlenecks for the widespread adoption of genome-edited crops. We propose a culture of sharing and integrating crop data to accelerate the discovery and prioritization of candidate genes, as well as a strong engagement with governments and the public to address environmental and health concerns and to achieve appropriate regulatory standards.

## Introduction

The vast amount of genomic data and the growing genome editing toolbox are key to the improvement of existing crops and the domestication of new crops [1–4]. In the past decade, the lower cost of DNA sequencing has allowed the assembly of more than 200 plant genomes [5], many of them crops and crop relatives. RNA sequencing and gene prediction algorithms have facilitated the annotation of these genomes [6]. Third-generation sequencing is further improving assemblies by moving them from scaffold-based draft genomes to chromosome-level reference assemblies [4], and resequencing is allowing the step towards pangenomes [7]. Within the next five years, a major sequencing and annotation effort hopes to generate more than 10,000 draft genome assemblies for plants [8]. Further, genome-wide association studies (GWAS) and quantitative trait loci (QTL) analyses are identifying substantial numbers of candidate regions that are linked to agronomic traits for use in crop improvement [9]. Nevertheless, phenotyping has lagged behind genotyping [10], and there is a divide in the amount of genotypic data available for model crops and for non-model crops and crop wild relatives. Closing the gaps between phenotypic and genotypic data and among the data for different crops and crop relatives will provide important information that will facilitate the widespread implementation of crop genome editing. Network analyses could then help to interpret this deluge of data to find agronomically relevant target genes [11].

Advances in genome editing now allow targeted mutation of crop genomes with base-pair precision using the CRISPR/Cas system [12]. The enormous potential of genome editing as a crop improvement tool has been highlighted in several recent reviews [1, 13–16]. The sharp rise in research interest in genome editing with CRISPR/Cas has led to innovative techniques for increasing the precision and efficiency of this system. Using ribonucleoprotein complexes, genome editing can be carried out without introducing exogenous DNA into cells [17, 18]. Further, the Cas12a protein improves flexibility in genome editing and base editing [19, 20], and the Cas13 protein has been rapidly established as an epigenome editing tool [21]. Gene knock-in methods, which are technically more challenging than gene knock-out methods, are also demonstrating higher success rates [22]. More than 50 computational tools have been developed to design the CRISPR/Cas guide RNA that is used to target Cas endonucleases to a genomic site (http://omictools.com/crispr-cas9-category) [23], including two aimed specifically at plants (CRISPR-P [24] and CRISPR-Plant [25]).

In the past two years, several genome-edited crops have entered the final stages of commercialization in the US [26], including an oilseed *Camelina sativa* crop with enhanced omega-3 oil, a soybean crop with drought and salt tolerance achieved by disrupting the *Drb2a* and *Drb2b* genes [27], and a waxy corn (*Zea mays*) with starch composed exclusively of amylopectin [28]. This corn crop was achieved by inactivating the endogenous waxy gene *Wx1*, which encodes a granule-bound starch synthase that catalyzes the production of amylose. In Canada, genome-edited varieties have been approved for five different crop types, with a total of 12 crop varieties either having been approved or nearing the end of the approval process [29]. Nevertheless, the regulatory status of genome-edited crops remains uncertain in many countries [30].

The bottlenecks for genome-edited crops are the discovery and prioritization of agronomic target genes [6] and how strictly governments choose to regulate these crops [31]. Although most major crop genomes have been sequenced and annotated, predicting phenotypes from genotypes is rarely possible, complicating target discovery. In addition, regulatory bodies around the world have the potential to limit the impact of this emerging technology, as discrepancy persists between the safety of genome-edited crops and the restrictions imposed by the laws that govern them [32]. In this article, we propose that addressing three important points will help to secure the future of genome-edited crops: 1) generate more open data for non-model crop species and crop wild relatives; 2) move towards data integration and network analyses to facilitate the discovery and prioritization of agronomic genes for editing; 3) engage governments to put in place a regulatory framework for genome-edited crops that addresses public and environmental health concerns without imposing unreasonable constraints.

## Bigger is better: Generating more open data for non-model crop species and crop wild relatives to fuel the search for genome editing targets

The discovery and prioritization of candidate genes are important first steps in the plant breeding pipeline [33]. Unlike conventional breeding or genomic selection, crop improvement using genome editing relies on candidate genes. The challenge in candidate gene prioritization is to integrate genome assemblies, functional annotations, phenotypes, genotypes, and the results of association studies. Annotated chromosome-level genome assemblies of 18 of the 20 most-produced crops worldwide [34] are publicly available via GenBank [5]. The two exceptions are sugar cane (*Saccharum* spp. hybrids) and onion (*Allium cepa*), both of which have notoriously complex and large genomes (> 10 Gb) that are still progressing towards high-quality assemblies. Recently, third-generation sequencing technologies providing long-range sequence data have begun to help crop genomes to move towards gold standard assemblies [4].

However, obtaining insights that are meaningful for crop genome editing from the available data depends on the detection of genes underlying agronomic traits. Important tools for the discovery of trait-gene associations are GWAS and QTL analysis, which leverage genome-wide variant data and the phenotypes of crop populations [9, 35]. The challenge in applying GWAS and QTL analysis for breeding is their limited resolution of candidate regions, which is often insufficient to pinpoint candidate genes [36]. GWAS can generally offer higher resolution than QTL analyses, but they rely on high levels of recombination that are rarely present in elite crop populations [36, 37]. Broadening the focus of trait association studies from model crops such as rice to non-model crops and crop wild relatives opens up an important source of plant diversity for breeding [38–40].

For genome editing to deliver on its promise of an accelerated plant breeding pipeline, the research community should work towards improved gene discovery and prioritization. Generating the right genotype and phenotype data to inform target gene discovery and gene prioritization is an important step towards streamlining crop genome editing. Although genotype data are widely available publicly for most crops, there is a lack of gold-standard diversity panels consisting of whole-genome sequences for hundreds of genetically diverse crop accessions. In addition, gold-standard diversity panels for crop wild relatives with high levels of historical recombination are needed to increase the resolution of GWAS [39]. Together with comprehensive genotype data, there is also a need for more of the associated phenotype data [10]. Manual phenotyping is costly and labor-intensive, but automated phenotyping can help generate more phenotype data for larger populations by increasing throughput and lowering labor costs [41, 42]. Making the germplasm and genotypes of gold-standard diversity panels available to the broader research community with high freedom to operate would be a major step forward for those seeking to detect trait–gene associations that can be targeted by genome editing.

Gene characterization informs gene prioritization for genome editing, but the vast majority of crop genes remain uncharacterized [6]. Characterizing a gene can be achieved experimentally or in silico using similarity-based computational methods. Full characterization of a gene provides a structured vocabulary in the form of gene ontology (GO) terms that are crucial for the efficient processing of large-scale annotations. Information on biological processes, molecular functions, and cell components systematically defines gene functions. However, because characterizing gene function is labor-intensive and costly, most efforts have focused on the model plant species *Arabidopsis thaliana* and, to a lesser extent, on rice. *Arabidopsis* gene annotations provide a useful starting point for assigning functions to crop genes on the basis of synteny, particularly in closely related crops such as *Brassica* species. Such comparative analysis is limited, however, by interspecific differences in gene content and function. Additional gene characterizations for non-model crops and crop wild relatives are therefore needed. High-throughput gene knock-out in crops is becoming more feasible [43, 44] and could help to increase the rate of gene characterization in crops.

In addition to generating novel data for gene discovery and gene prioritization, the opening of currently closed data would immediately benefit the crop science community. Both within industry and academia, substantial amounts of genotypic and phenotypic crop data, including the results of genome-editing experiments, are closed to the wider community to protect intellectual property and because of the lack of dedicated data repositories [45]. In particular, the results of the early stages of gene discovery in breeding pipelines in industry are likely to be closely guarded. This may lead to widespread redundancy in research and development between competing industrial laboratories, increasing costs for companies. To address the issue of closed crop data and potentially wasteful competition, government funding can promote collaboration with industry and require the publication of a reasonable amount of the results. Incentivizing the publication of early discovery research through tax breaks and encouraging companies to view early candidate gene discovery research as pre-competitive may also spur data sharing with the wider community [46]. The public sector should set an example of openness by driving data-sharing initiatives across universities and other public research organizations.

## Integrating data and moving towards network analyses to identify candidate genes

Different types of ‘omic’ and phenotypic evidence must be brought together for the large-scale detection of crop genome-editing targets. Generic sequence repositories such as GenBank [47] and the European Molecular Biological Laboratory [48], as well as plant-specific repositories such as PlantGDB [49] and Phytozome [50], store genomic data without integrating proteomic, variant, or phenotype data from other sources. In addition, genomic data on CRISPR/Cas genome-editing experiments in plants showing cleavage activity and guide RNA efficiency are often not systematically integrated. For selected major crops, some of these gaps in generic repositories are being filled by specialized databases, for example, SoyBase [51], Grain Genes [52], and T3 wheat [53]. Additional work is being conducted in wheat and rice to develop single information systems that integrate large amounts of the available resources [54]. These databases bring together annotated genome sequences, genetic maps, genetic variants, gene functions, gene expression, interaction networks, pedigree data, and trait information. Nevertheless, they still do not encompass all the available dispersed data including genome editing experiments, and for many crops integrated databases do not yet exist.

Network analysis can help to bring together heterogeneous data types to allow non-hypothesis-driven queries for trait-associated target genes [55]. These queries help to generate useful candidates for genome editing in the discovery stages of the plant breeding pipeline. For example, most gene function prediction depends on guilt-by-association methods that are based on gene expression [55]. By leveraging protein–protein interactions, literature text mining, coexpression, genomic-neighbor information, gene function, and domain co-occurrence, a cofunction network that provided prediction accuracy higher than any single method was constructed in *Arabidopsis* [56]. The inclusion of phenotype data in network analyses has also been shown to increase the effectiveness of gene prioritization [57]. These data help address the challenges in GWAS and QTL analyses of linkage disequilibrium between associated variants and lack of functional annotation, which often cause these studies to fall short of finding causal variants. By applying a meta-analysis with multiple inference methods to studies of gene cofunction, prediction accuracy can be further improved [58], although more inference methods may not always increase performance linearly [55].

Integrative network analyses to prioritize candidate genes are becoming more frequent in mammalian systems [57, 59–61], but they are still rarely implemented in plants, with exceptions in *Arabidopsis* [62]. A step forward for crops may be the intelligent mining of dispersed data networks. For instance, KnetMiner (Knowledge Network Miner) is a web tool designed for gene discovery using diverse biological data including literature [11]. KnetMiner ranks genes for associations with traits on the basis of network analyses. For example, KnetMiner found an association between the barley gene MLOC_10687.2 and seed width [63]. More comprehensive use of network analyses in crop gene discovery and gene prioritization will ensure the availability of genome-editing targets for a range of agronomic traits.

## Moving from a global patchwork of crop biotechnology regulation towards product-based regulation

Restrictive regulation of genome-edited crops could limit the future impact of these crops on agriculture. Globally, genome-edited crops are currently regulated with either process-based or product-based approaches, although in some countries the regulatory concepts remain unclear [30]. Process-based regulations focus on the biotechnological processes that are used to alter crop DNA. Conversely, the product-based approach regulates the resulting crop plant and its traits, not the breeding process used to create it [64].

Most crop biotechnology regulatory frameworks were developed or updated to regulate genetically modified organisms (GMOs). GMOs include exogenous DNA that would rarely be present through natural processes. This use of exogenous DNA has raised public and environmental health concerns, resulting in strict regulations in many countries. Many genome-editing approaches do not, however, lead to the presence of exogenous DNA in the final plant product [65]. Indeed, genome editing with ribonucleoprotein complexes avoids the introduction of any exogenous DNA during the breeding process [66] and base-editing techniques do not even require cleavage of DNA [67]. Despite this important difference between GMOs and genome-edited crops, they may be regulated similarly on the basis of the breeding process.

In an analysis of regulatory concepts in 33 countries and the EU, including 24 countries in which GM crops are commercially cultivated, it was found that 15 countries and the EU used process-based regulations and 14 countries use product-based regulations [30]. Four countries (Paraguay, Myanmar, Chile, and Vietnam) did not have a clear regulatory framework. Among large agricultural producers, Argentina, Canada, the Philippines, and Bangladesh use product-based regulations, whereas Brazil, India, China, Australia, the EU, and New Zealand use process-based regulations. The EU regulates any crop that has undergone genetic editing as a GMO [68]. Within the EU, GMOs are defined as “organisms in which the genetic material (DNA) has been altered in a way that does not occur naturally by mating or natural recombination” [69]. These regulations extend to genome-editing involving the transient use of recombinant DNA that does not lead to transgenes in the final product. In early 2018, the Advocate General of the Court of Justice of the EU (CJEU) suggested that crops developed with genome editing without using recombinant DNA may not be regulated as GMOs, indicating the move towards a product-based assessment [70]. However, a recent ruling by the CJEU has classified genome-edited plants as GMOs [71]. The CJEU made this decision based on the consideration that genome editing “alter[s] the genetic material of an organism in a way that does not occur naturally”, concluding that the associated risks may be similar to those posed by GMOs [72].

In contrast to the EU, Canada regulates the final plant product, irrespective of the process used to produce it [73]. The safety of the crop is determined by the presence of a novel trait, which is defined as “a trait which is both new to the Canadian environment and has the potential to affect the specific use and safety of the plant with respect to the environment and human health.” These traits can be introduced using genome editing, mutagenesis, or conventional breeding techniques [74]. Similarly to Canada, the US assesses biotechnology products on a case-by-case basis relying on the Coordinated Framework for Regulation of Biotechnology [73], with regulation carried out by the Food and Drug Administration (FDA), the Environmental Protection Agency (EPA), and the US Department of Agriculture (USDA). The Coordinated Framework for Regulation of Biotechnology was completed in 1986 and dictates that only the final plant product can be subject to regulation and that biotechnological process will be assumed to be safe unless there is appropriate scientific evidence suggesting otherwise. Nevertheless, the US adopts a less stringent product-based approach than Canada [75]. For instance, regulation by the USDA is triggered when a plant pest is used as transformation vector or DNA donor, which is the case for most GM crops transformed using the widespread *Agrobacterium* vector. Regulation can also be triggered when a plant expresses a pesticide trait (EPA regulation) or poses food safety risks (FDA regulation). The USDA has signaled that it does not regulate transgene-free genome-edited crops that do not pose a plant pest risk [26, 76], and the EPA and FDA have not commented on their regulatory role for these crops. In June 2018, however, the USDA issued a notice of intent, indicating that it was considering updating its biotechnology regulations in response to advances in genetic engineering [77].

Last, Argentina also adopts a largely product-based approach to genome-edited and genetically modified crops. Unlike most biotechnology regulation, Argentina’s regulation was specifically designed to accommodate new breeding techniques. A central concept in their case-by-case assessment of organisms is the presence of “novel combinations of genetic material”, which determines whether or not an organism is regarded as a GMO [78]. Argentina’s legislation also allows for flexibility of new technologies, as there is no clear-cut definition of breeding processes that are included in the legislation. This allows for the legislation to be applicable to a variety of new breeding techniques that are likely to arise in the future [78]. It is essential for regulation to reflect the differences between GMOs and genome-edited crops, as these technologies are significantly different and their products have the potential to play an important role in food security, particularly in developing countries [12].

Although there are many benefits to incorporating genome editing in plant breeding [15], public perception plays a large role in the commercialization of biotechnology [79]. GMO food products have lacked widespread public approval in some countries because of their novelty and perceived negative health effects, which could also affect the public image of genome-edited crops [80]. Concerns held by the public can put pressure on government bodies to restrict the application of agricultural biotechnology and to limit scientific innovation [81]. Therefore, scientists, the media, and regulatory bodies should place emphasis on engaging the public in factual discussions regarding the safety of genome-editing. Genome-edited crops could increase consumer acceptance of biotechnology in agriculture because of the lack of transgenes, which are the public’s primary concern [82]. There is also a strong need for more transparent legislation that can accommodate current and future plant-breeding techniques. For instance, the CJEU ruling [72] does not fully resolve the issue of genome-edited crops in the EU as it applies only to nuclease- or nucleotide-directed mutagenesis techniques. Further, the ruling is inconsistent with the regulatory exemption for chemical and radiation mutagenesis techniques, as these techniques are widely considered to have similar or higher levels of risk compared to genome editing [83]. Updates to biotechnology regulation in the EU and elsewhere should therefore be seen as an opportunity to develop fit-for-purpose and consistent regulation for rapidly advancing technology [84]. Suggestions for a novel regulatory framework have been put forward, emphasizing careful documentation of all genetic changes made in a new product [85]. Importantly, any potential risks of genome editing should be evaluated alongside the benefits that the technology is likely to bring. This approach will prevent policies that are unnecessarily risk-averse from restricting the advancement of biotechnology research and commercialization.

## Abbreviations

CJEU: Court of Justice of the EU
EPA: Environmental Protection Agency
FDA: Food and Drug Administration
GMO: Genetically modified organism
GWAS: Genome-wide association study
KnetMiner: Knowledge Network Miner
QTL: Quantitative trait loci
USDA: US Department of Agriculture
